# Coastal business perception of coral value and payment for coral restoration

**DOI:** 10.1038/s41598-025-93439-x

**Published:** 2025-03-18

**Authors:** Rachel R. Carlson, Joanna Klitzke, Gretchen C. Daily, Larry B. Crowder, Borja G. Reguero, Gregory P. Asner

**Affiliations:** 1 Emmett Interdisciplinary Program in Environment and Resources, Stanford University, Stanford, CA USA; 2 Stanford Graduate School of Business, Stanford University, Stanford, CA USA; 3 Center for Global Discovery and Conservation Science, Arizona State University, Hilo, HI USA; 4 Hopkins Marine Station, Stanford University, Monterey, CA USA; 5 Department of Biology, Stanford University, Stanford, CA USA; 6 Natural Capital Project, Stanford University, Stanford, CA USA; 7 Woods Institute for the Environment, Stanford University, Stanford, CA USA; 8 Coastal Science and Policy, University of California, Santa Cruz, Santa Cruz, CA USA; 9 Department of Environmental Science, Policy, and Management, University of California, Berkeley, CA USA

**Keywords:** Coral, Ecosystem services, Tourism, Restoration, Nature-based solutions, Coastal resilience, Environmental sciences, Conservation biology

## Abstract

Coral reefs provide important economic benefits to coastal businesses, supporting recreation and tourism and protecting property from storms. Yet, these benefits are at risk worldwide as corals decline rapidly, and investment in restoration is lacking. With their direct dependence on coral health, coastal businesses may represent an important sector for funding coral restoration; however, it is unclear whether businesses perceive coral reef services as valuable or themselves as reef stewards. We measured business perceptions of coral health and value in Hawaiʻi and identified traits correlated with business decisions to participate in coral restoration at three payment thresholds. We found that businesses see limited economic value in coral reefs. In areas where corals provide substantial ecosystem services (flood protection, tourism revenue), businesses did not consistently rate coral value as high. Nonetheless, businesses showed strong willingness to pay for coral restoration, which was linked to pro-nature motives, reputation, and Native Hawaiian ownership. Results highlight key strategies for engaging private entities in coral restoration.

## Introduction

Coral reefs are increasingly recognized as economic assets, providing critical ecosystem services that sustain commercial activity along coastlines^1,2^. Coral reefs provide storm protection for at least 100 million coastal residents worldwide, reducing wave energy during large storms by 97% and mitigating storm damage at levels similar to built infrastructure like breakwaters or levees^3^. In the U.S., a recent resolution (USCRTF Resolution 47.2) designates coral reefs as national natural infrastructure based on the coastal protection benefits they provide^4^. These benefits are worth over $1.8 billion annually across the 50 US states, with further protection provided in US Territories^5^. Moreover, corals generate economic benefits in the form of recreation, tourism, and food security. Non-risk reduction services of coral reefs in the US have been estimated at US$3.39 billion annually^6^; for example, tourists in Hawaiʻi expressed willingness to pay $15.33–20.22 person^−1^ day^−1^ to improve coral health for recreation^7^.

As the private sector and especially the tourism industry significantly benefit from flood mitigation^8^ and other ecosystem services^6^ provided by coral reefs, this sector represents an important but largely untapped^9^ stakeholder to contribute to coral restoration. Coastal businesses like hotels, retail stores, restaurants, and ocean recreation companies receive direct revenue from tourists attracted by coral reefs and by ocean activities that depend on coral reef habitat, like fishing and surfing^10^. In a global assessment of coral reef tourism values, Spalding et al.^11^ classified reef benefits to coastal businesses as either “reef-adjacent” or “on-reef,” where “on-reef” benefits include revenue from diving or snorkeling, while less obvious “reef-adjacent” benefits include tourist spending on amenities that are indirectly linked to coral reefs such as sandy beaches, calm water, shoreline stability^12^, food^13^, surf breaks^14^, and coral reef imagery used in marketing^15^. Importantly, corals protect sandy beaches from erosion, and beaches represent one of the primary criteria for tourists selecting lodging and recreation sites^12,16^. In total, direct and indirect benefits of coral reefs to tourism are valued at $36 billion per year worldwide^11^. Given increasing climatic and local threats to coral reefs and their high economic benefits for the tourism industry, a strong incentive may exist for coastal businesses to contribute to coral restoration strategies.

Conservationists have increasingly turned to the private sector to invest in coral reef conservation and restoration, particularly as the public sector has failed to adequately fund environmental protection in many regions^17^ while threats to reefs such as climate change intensify. However, to date, private-sector financing models for coral reef protection remain experimental and underutilized. One method of coral reef financing that has gained recent attention is parametric insurance. In a parametric insurance model, coastal stakeholders like hotel owners and marine operators purchase an insurance policy that pays out for coral restoration after natural disasters. Parametric insurance is based on a predetermined threat or “parameter” threshold (e.g., windspeed or ocean temperature) that is reliably linked to coral damage. If a disaster like a hurricane or heatwave drives the parameter past the predetermined threshold, (e.g., 100 mph windspeed), this triggers an insurance payout^18^ that funds coral restoration like coral outplanting or removing marine debris after a storm. Parametric insurance has been increasingly used to protect coral reefs^19–21^. For example, the hotel industry in Mexico was critical in funding a coral insurance pilot in Quintana Roo^18^, where corals provide a value of 20 million USD to hotels per year^22^, and the same model was implemented in a pilot program in Hawai’i in 2023 led by The Nature Conservancy^21^. However, it is unclear whether and which types of businesses would be willing to invest in insurance for coral reefs, or otherwise fund reef restoration. For such programs to be scalable, additional information is needed about drivers of business participation in coral restoration specifically, and financing natural capital more generally.

Drivers of private-sector participation in coral restoration are largely unstudied. One uncertainty is business perception of coral value. Coastal businesses may not acknowledge the ecosystem services provided by coral reefs and view nature simply through the lens of negative externalities^23^. Natural capital is often viewed in isolation from traditional forms of economic and social capital^24^ and, therefore, is often neglected in decision making. Nonetheless, several case studies have highlighted business leadership in reef conservation. For example, Bottema and Bush^25^ found evidence of “environmental entrepreneurs,” e.g., private companies that create social capital by spearheading ocean conservation initiatives. Nonetheless, such cases may represent exceptions rather than industry-wide norms. Furthermore, many studies have evaluated tourists and recreationalist willingness to pay (WTP) for recreational experiences on protected or healthier coral reefs, e.g.^7,26^; however, these studies focus primarily on tourists and not private entities themselves. Coastal business are intermediaries between tourists and reefs, often setting the price that individuals pay for reef experiences, and it is therefore critical to evaluate whether businesses perceive corals as an economic asset. Furthermore, it is unknown which types of businesses assign high economic value to coral reefs; whether perceived value differs between areas with high and low ecosystem services; and between industries operating directly on the reef (e.g., scuba shops) versus more distantly (beachside restaurants, hotels).

To assess business perceptions and willingness to support coral restoration, here we surveyed 202 coastal businesses on two Hawaiian Islands. Hawai’i is the site of a new parametric insurance policy for coral reefs that, at the time of data collection, was under development and has since been implemented^21^; our study was thus designed to be useful to a realistic insurance pilot. We first measured the extent to which coastal businesses perceive coral reefs as valuable across varying industries and levels of ecosystem services, specifically flood protection, recreation, and tourism. In parallel, we analyzed how businesses rate coral health across different industries and levels of coral cover measured using remote sensing^27^. Finally, we typified which businesses show early support for coral restoration by voicing willingness to buy into coral insurance at three optional payment thresholds ($${\raise.5ex\hbox{$\scriptstyle 1$}\kern-.1em/ \kern-.15em\lower.25ex\hbox{$\scriptstyle 8$} }$$, ¼, and ½ of 1% of annual revenue, converted to a dollar value). We assessed possible drivers of buy-in such as business’ structural features (e.g., size of company), financial motivations (perception of coral value to business operations), and two additional, though preliminary, indices derived from behavioral economics^28^: regard for business reputation and intrinsic motivation (protecting corals because they are inherently worthy). The analysis provides insights into the planning and limitations of coral reef financing tools targeting the private sector and suggests next steps including future research to catalyze voluntary restoration efforts in the tourism sector.

## Results

We surveyed 202 businesses representing 259 land-based or shoreline locations and 52 unique ocean operating locations. We removed six respondents from the dataset that were missing responses to over five survey questions, analyzing data from 196 respondents (responses summarized in Supplementary Table S3). A total of 21.1% of respondents replied to the survey via email while 78.9% of respondents replied in person. 19 and 87% of respondents who were approached, respectively, via email and in person agreed to complete the survey. Most business owners were long-time or generational residents of Hawaiʻi, as 55.2% lived on island for > 20 years and 19.6% were Native Hawaiian. Respondents were evenly distributed between age groups and genders and were typically higher-level managers or full owners of businesses with 65.4% reporting significant or full control over business budgetary decisions. Respondents were not included if they reported “no control” over budgetary decisions.

Due to higher business density on Oʻahu, the majority (71.4%) of respondents were based on Oʻahu, primarily Waikīkī, Kāneʻohe Bay, Kailua Bay, Hanauma Bay, North Shore/Haleʻiwa, and Mākaha. 26.0% of respondents were based on Hawaiʻi Island, with higher concentrations in Hilo, Kealakekua, and Kailua-Kona. 84.0% of businesses surveyed earned > $100,000 in revenue per year, with the total survey population representing a maximum net annual revenue of $511 M, though the maximum revenue of the highest-earning businesses (survey response of “ > $10 M per year”) was unspecified. Recreational businesses were evenly distributed between surface-oriented recreation (surf, kayak, and boating = 42 businesses) and subsurface recreation (dive, snorkel, and spearfishing = 37 businesses), while shoreline business included retail (clothing, galleries, specialty goods like natural products = 86 businesses), restaurants (22 businesses), and lodging (7 businesses).

### Perception of coral health

**Fig. 1:**
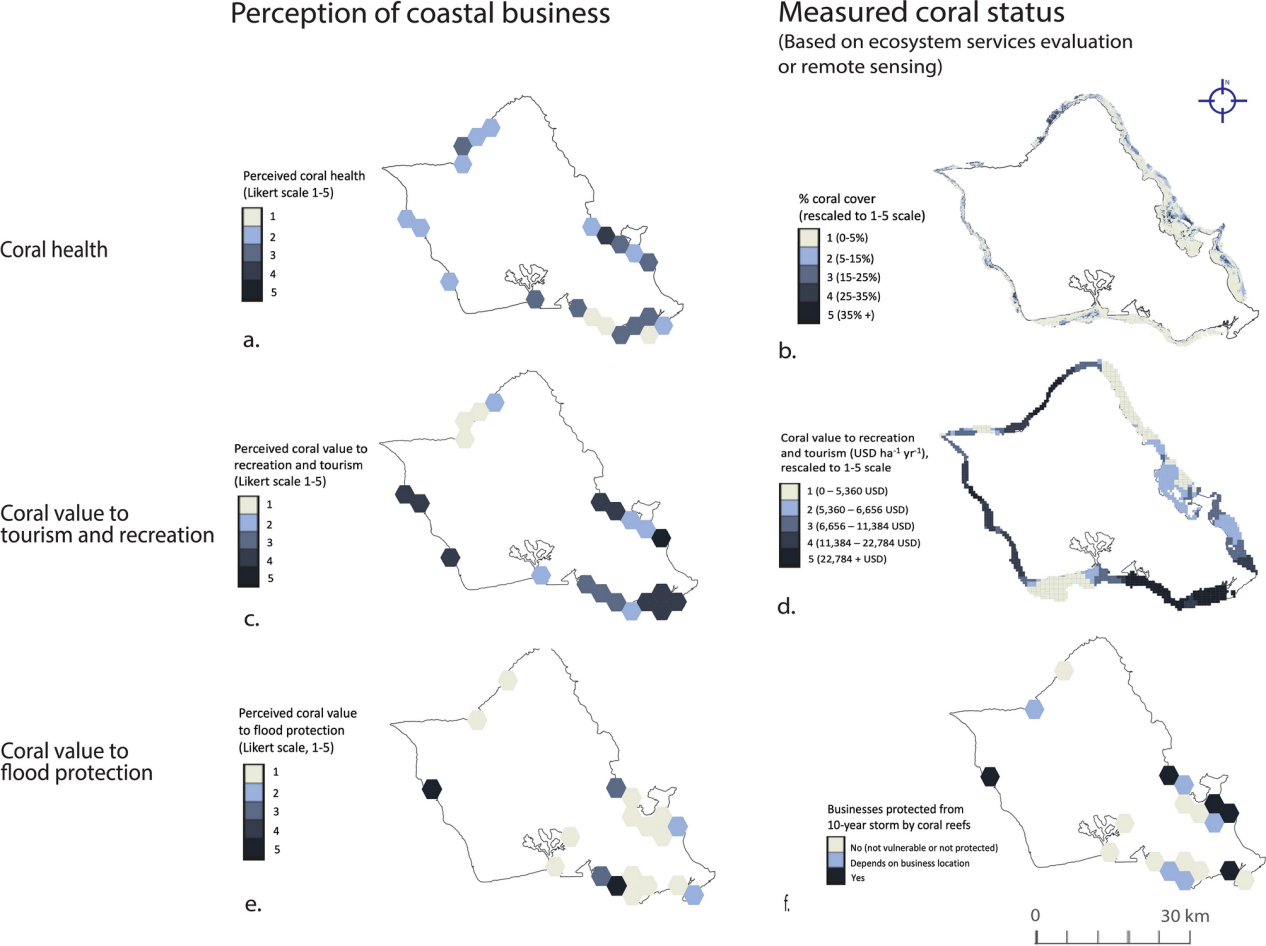
Map of Oʻahu showing that businesses overestimate coral cover and underestimate ecosystem services. (**a**) Business perception of coral health compared to remotely sensed coral cover, where both are compared on a 1–5 scale. In most regions, perceived coral health is higher than remotely sensed^27^ coral cover. (**b**) Business perception of coral value to recreation and tourism, compared to Mapping Ocean Wealth^11^ data on coral value to recreation and tourism (USD ha^−1^ year^−1^), rescaled to a 1–5 scale. Businesses underestimate reef value to tourism/recreation in regions like Honolulu and North Shore/Haleʻiwa. (**c**) Business perception of coral value to flood protection v. areas where corals confer flood protection (yes/no) in a 10-year storm^8^. Businesses underestimate coral value to flood protection in many regions of Oʻahu relative to flood projections^8^.

On average, businesses reported coral health of 2.4 ± 0.94 (between “Unhealthy” and “Fair”) on a 5-point scale. Mean remotely sensed coral cover surrounding businesses ranged from 0 – 33%, which converted to a mean of 2.8 ± 0.9 when rescaled to a 5-point scale using intra-island quintiles (see Methods). Rescaled coral cover does not equate directly to ecosystem status (the highest quintile of remotely sensed coral cover is not necessarily a “Very Healthy” reef), nor do we expect unique individuals to have equivalent definitions of a “Very Healthy” reef. Nonetheless, our respective indices suggest that, on average, businesses reported reefs as “Unhealthy” to “Fair” while operating near reefs in middle quintiles of coral cover. However, when remotely sensed coral cover was rescaled based on a historic, 20-year baseline (Methods), coral reefs surrounding businesses had an index of 1.2 ± 0.4. Based on the latter rescaling method, remotely sensed coral cover was low (1–2 on 5-point scale) in many regions across Oʻahu and Hawaiʻi where businesses perceived coral cover as “Fair” to “Very Healthy” (Fig. 1; Supplementary Fig. S1). For example, in Waikiki, many businesses ranked coral health as “Fair” despite 0–5% coral cover. Only select dive destinations near Kona and North Shore (e.g., Shark’s Cove) showed the inverse pattern, i.e., businesses estimating coral health as “Unhealthy” despite remote sensing showing highest-quintile coral cover.

**Fig. 2:**
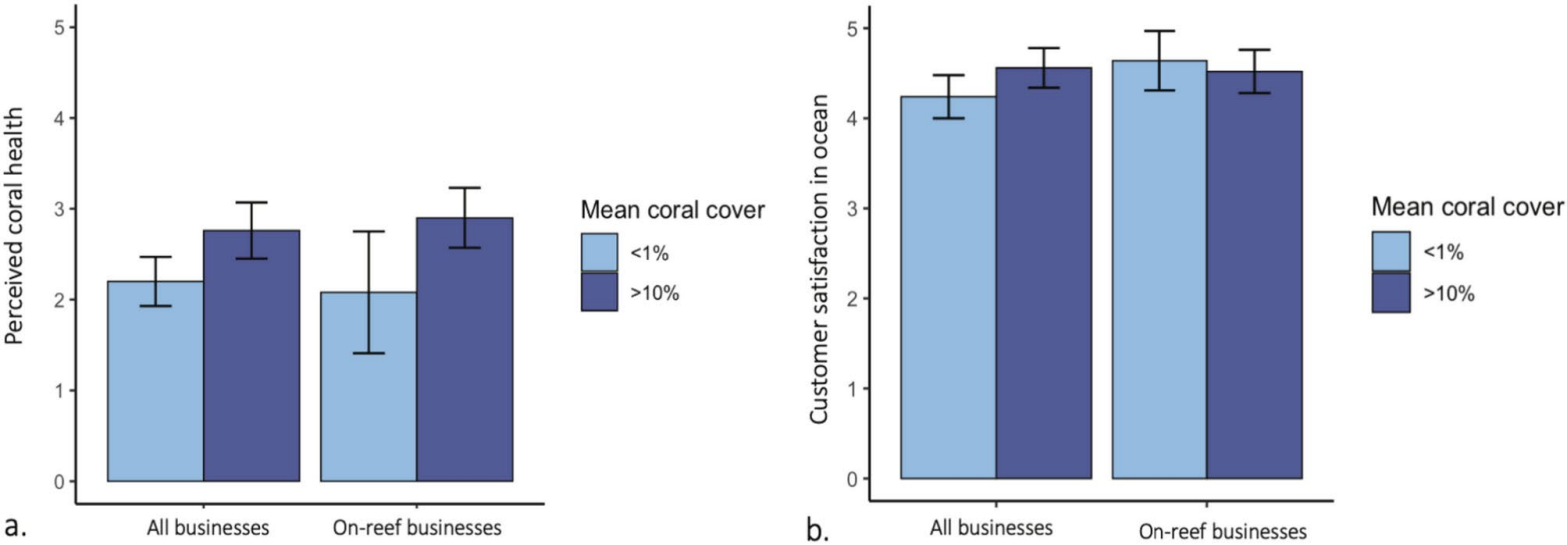
There is no significant difference in perception of coral health between businesses in low and high coral cover areas. (**a**) Mean perceived coral health (± 95% confidence interval) and (**b**) Mean customer satisfaction in low and high coral cover areas. X-axis displays perception of all businesses (left x-axis), and only on-reef businesses (right x-axis) such as dive and snorkel companies. Confidence intervals were generated using bootstrapping; overlapping confidence intervals indicate no significant differences between groups.

There was no significant difference in perceived coral health among businesses located in low- and high- coral cover areas (ʻlow coral cover’ < 1% and ‘high coral cover’ = 10–24% mean cover). Businesses near high coral cover reported coral health of 2.7 ± 0.3 compared to 2.2 ± 0.3 in low-cover areas, rated on a 5-point Likert scale (Fig. 2a). Similarly, while remote sensing showed higher mean coral cover on Hawaiʻi Island (7.5 ± 2.1%) compared to Oʻahu (3.4 ± 0.6%), there was no significant difference between perceived coral health per island (2.3 ± 0.2 on Oʻahu; 2.6 ± 0.3 on Hawaiʻi Island). Finally, among different industries, there were no significant differences in perceived coral health. Note that, for marine operators (e.g., snorkel charters), we evaluated remotely sensed coral cover at the ocean locations they visit with customers, whereas shoreline businesses (e.g., restaurants) were evaluated for coral cover within a buffer of their shoreline location (see Methods).

‘Perceived customer satisfaction in the ocean’ did not significantly differ between companies that were surrounded by low and higher coral cover, even for businesses operating directly on the reef like dive and snorkel companies (Fig. 2b). Ocean recreation businesses operating in little to no coral reef (< 1% mean cover) reported mean customer satisfaction in the ocean of 4.2 ± 0.2, while ocean recreation businesses operating in 10–24% mean coral cover reported customer satisfaction of 4.5 ± 0.2. Almost all businesses agreed with the statement, “My customers are generally satisfied with their experience in or around the ocean” regardless of remotely sensed coral cover nearby (Fig. 2b). Only one business disagreed with this statement, and the mean response was 4.3 ± 0.9 (between “Agree” and “Strongly agree”).

### Perception of coral value: recreation and tourism

**Fig. 3:**
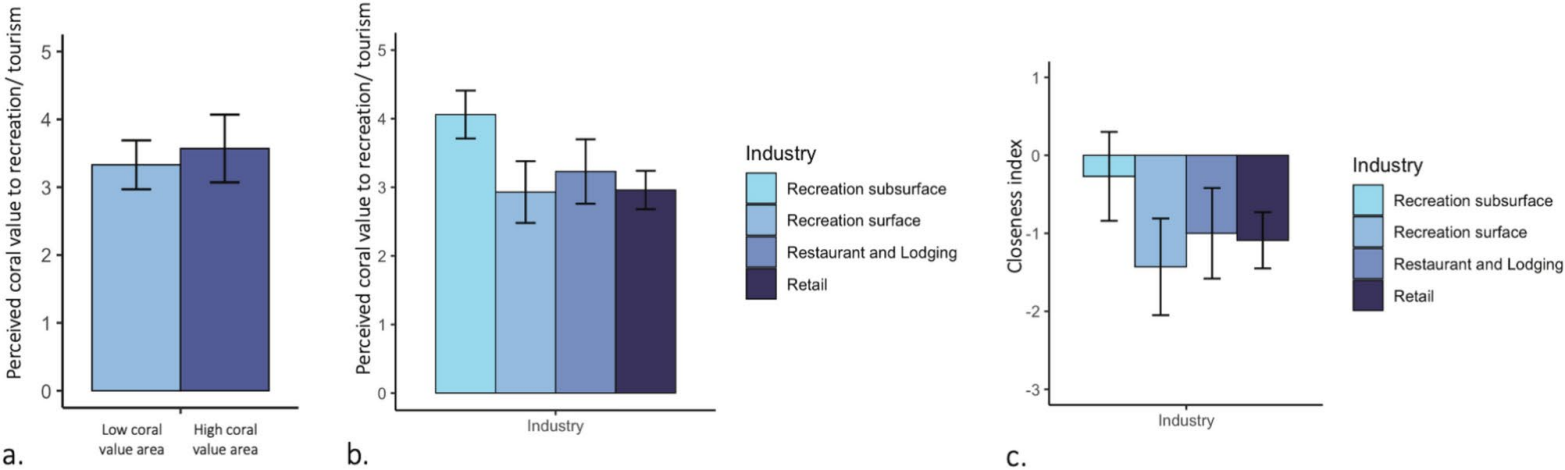
Businesses do not rank coral value significantly higher in areas where corals provide high v. low recreation and tourism services, based on a widely used ecosystem services dataset (Mapping Ocean Wealth; MOW^11^). (**a**) Mean (± 95% confidence interval) perceived coral value to tourism in the lowest v. highest coral value regions based on MOW, (**b**) Perceived coral value to tourism across different industries, (**c**) Mean ‘closeness index’ (similarity between business- and MOW-ranked coral value) by industry. Confidence intervals were generated using bootstrapping; overlapping confidence intervals indicate no significant differences between groups.

Perceived coral value differed across industries, as recreational businesses that operate below the surface (dive and snorkel operations) reported significantly higher perceived reef value (4.1 ± 0.4; Fig. 3b) than other industries. Importantly, recreational businesses operating on the ocean surface (kayak, surfing, and other watersports) valued coral reefs the lowest of all sectors (2.9 ± 0.5), with ratings similar to the retail sector.

Businesses ranked coral value as, on average, “Fair” while operating near reefs in high quintiles of a widely used dataset quantifying tourism/recreation services (Mapping Ocean Wealth; MOW^11^; Fig. 1; Supplementary Fig. S1). Businesses reported a mean coral recreational value of 3.2 ± 1.3 on a 5-point scale, while MOW ecosystem services data estimated coral recreational value at 4.2 ± 1.0 after rescaling to a 5-point scale (see Methods). Non-rescaled MOW coral recreational value per hectare ranged from 5600–507,000 USD ha^-1^ year^−1^.

Business perception of coral value did not significantly differ between regions with the lowest and highest coral ecosystem services in tourism/recreation. In regions with the lowest and highest 20th percentile of MOW coral value to recreation/tourism, businesses rated coral value as 3.3 ± 0.4 and 3.6 ± 0.5, respectively, on a 1–5 Likert Scale (Fig. 3a).

### Perception of coral value: flood protection

**Fig. 4:**
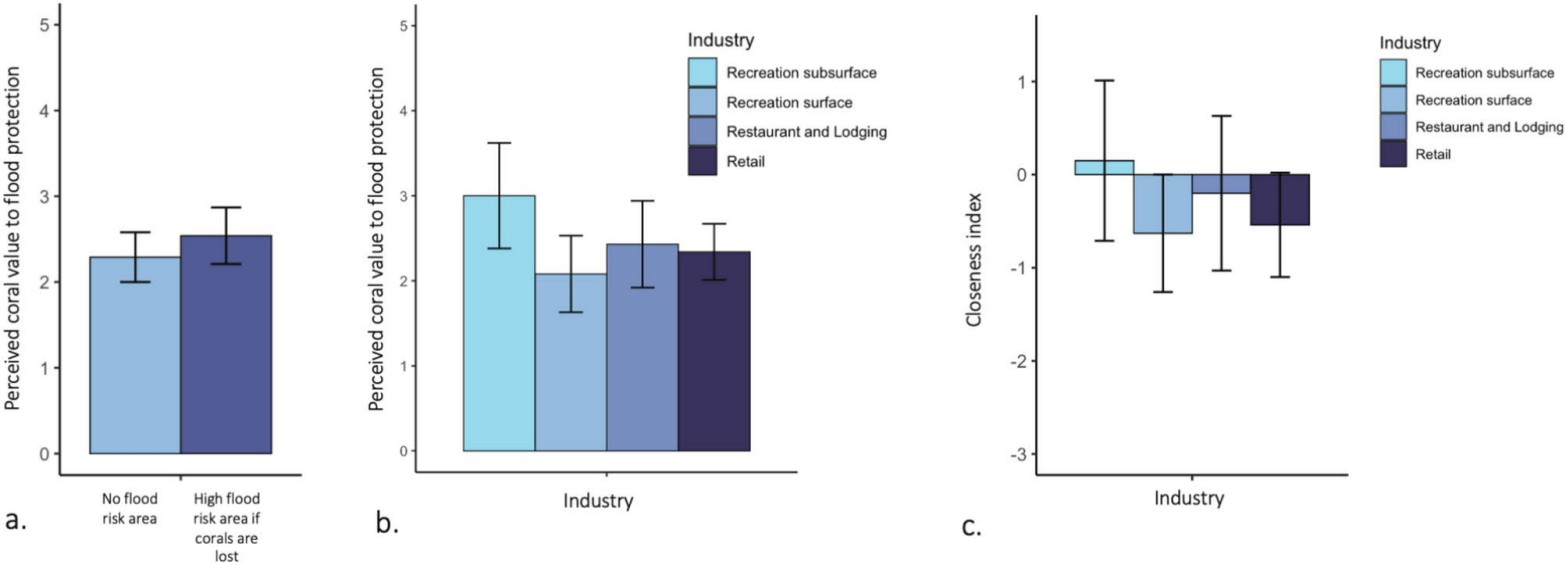
There is no significant difference in business perception of coral value in areas where corals offer high v. low flood protection, based on 10-year floodplain projections^8^. (**a**) Mean (± 95% confidence interval) perceived coral value to flood protection in areas with high v. low flood protection by coral reefs, (**b**) Perceived coral value to flood protection by different industries, (**c**) ‘Closeness index’ (similarity between business-ranked coral value and flood projections) by industry. Confidence intervals were generated using bootstrapping; overlapping confidence intervals indicate no significant differences between groups.

If the top-most 1 m of coral reef is lost in the future, 114 of the 259 land-based facilities (44.3%) in our dataset are expected to experience increased flooding in a 10-year storm. Survey responses did not significantly differ between flood-prone and flood-safe businesses when asked whether coral loss would put their physical infrastructure at risk (2.5 ± 0.3 and 2.3 ± 0.3 on a Likert scale, respectively; Fig. 4a). The average business in Oʻahu underestimated coral value to flood protection relative to floodplain models (closeness index of −1.0 ± 0.4; Fig. 1; see Methods), while the average business in Hawaiʻi Island overestimated coral value to flood protection (closeness index of 1.2 ± 0.5; Supplementary Fig. S1).

When comparing industries, there were no significant differences between industries in perceived coral value to flood protection (Fig. 4b-c). However, subsurface recreation companies and coastal restaurants/lodging had a closeness index approaching zero. In other words, this industry matched floodplain models^8^ in rating whether corals confer protection.

### Payment for coral insurance

We assessed business participation in coral restoration as a binary variable (yes/no willing to participate) at three buy-in thresholds, based on the scenario of coral insurance described in Supplementary Fig. S3 and with design elements adapted from previous case studies and associated principles of parametric insurance^18–21^. We also assessed previous donations of staff time or funding to coral restoration (binary yes/no), hereafter referred to as “revealed preference” (see Methods). Most respondents were willing to participate in coral insurance: 68.7% of businesses responded “Yes” to at least the minimum payment of 0.125% of annual revenue (Supplementary Table S3). The most common choice of payment was the maximum payment possible in the survey, i.e., 0.5% of annual revenue. However, 56 businesses or 31.3% were unwilling to participate in coral insurance; common reasons for non-participation are noted in the Discussion.

**Fig. 5:**
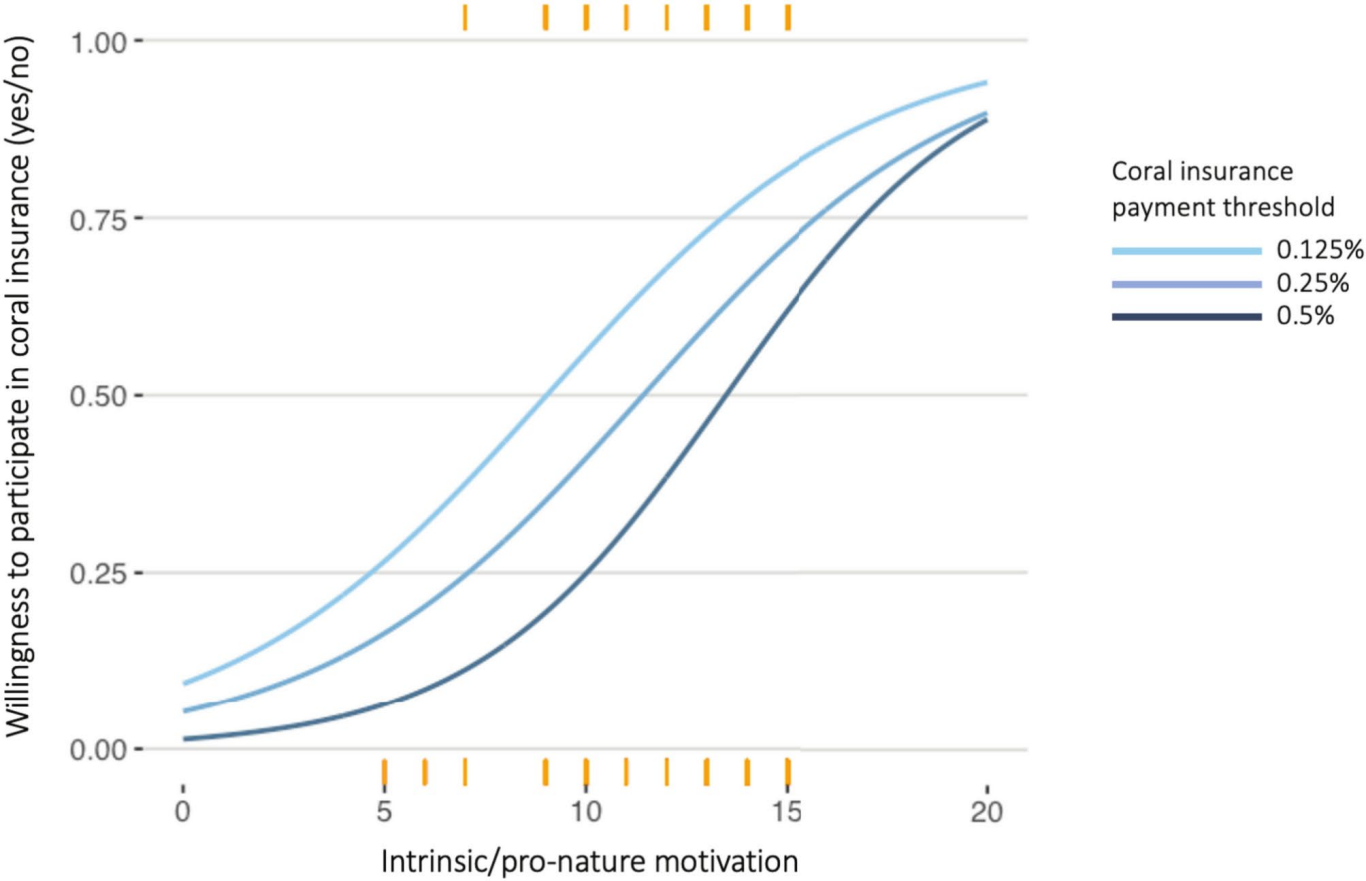
Relationship between intrinsic/pro-nature motivation and probability of participating in coral reef restoration through the scenario of insurance. Lines denote participation probability at three distinct payment thresholds ($${\raise.5ex\hbox{$\scriptstyle 1$}\kern-.1em/ \kern-.15em\lower.25ex\hbox{$\scriptstyle 8$} }$$, ¼ and ½ of 1% annual revenue); orange lines represent distribution of survey responses along intrinsic/pro-nature motivation scale (x-axis) and corresponding willingness to participate in program at three set payment thresholds (1 = yes to payment, 0 = no to payment; y-axis).

Material benefits, i.e., perceived value of coral reefs to business operations, were not significantly correlated with participation at any payment threshold. In contrast, ‘intrinsic (pro-nature) motivation’ was strongly correlated with participation at every payment threshold, ranging from *β* = 0.2 ± 0.1 to *β* = 0.3 ± 0.1 *p* < 0.0 (Fig. 5; Table 1). This equates to an odds ratio of 1.2 – 1.4; in other words, the odds of participation is 1.2–1.4 times higher given a 1-unit increase ‘intrinsic (pro-nature) motivation.’ Native Hawaiian ethnicity was also highly significant and positive in two of the models (*β* = 1.6 ± 0.6, *p* < 0.05 at 0.25% payment threshold and *β* = 2.0 ± 0.7, *p* < 0.01 at 0.5% threshold), equating to an odds ratio of 5.0 and 7.4, respectively. In other words, if the respondent was Native Hawaiian, they were 5–7.4 times more likely to participate. The majority of Hawaiian business owners or managers surveyed were willing to pay for coral insurance at the highest buy-in threshold of 0.5% of annual revenue (Supplementary Table S3; Supplementary Fig. S2b). For all significant variables, as the payment threshold offered by the survey increased, the effect size (regression coefficient) of variables also increased.

**Table 1:** Drivers of willingness to participate in coral insurance, modeled for three payment thresholds (bid levels). Regression coefficients shown alongside standard error in parentheses. Results of “revealed preference” model can be found in (Supplementary Table S4).

	Participation at $${\raise.5ex\hbox{$\scriptstyle 1$}\kern-.1em/ \kern-.15em\lower.25ex\hbox{$\scriptstyle 8$} }$$ of 1% of revenue		Participation at ¼ of 1% of revenue		Participation at ½ of 1% of revenue	
	All variables	Best fit	All variables	Best fit	All variables	Best fit
Distribution method (effect shown: in person)	1.138 (0.605)	–	0.477 (0.562)	–	−0.159 (0.598)	–
Years in Hawaiʻi	0.116 (0.177)	–	0.131 (0.164)	–	0.067 (0.164)	–
Identity/ethnicity (effect shown: Hawaiian/indigenous)	1.447 (0.782)	1.192 (0.738)	1.657* **(0.683)**	1.601* **(0.642)**	2.003** **(0.720)**	2.005** **(0.690)**
Age	−0.181 (0.269)	–	0.073 (0.246)	–	0.065 (0.258)	–
Gender (effect shown: Male)	−0.190 (0.422)	–	−0.279 (0.382)	–	−0.227 (0.397)	–
Island (effect shown: Oʻahu)	−0.830 (0.551)	–	−0.050 (0.471)	−0.149 0.403	−0.215 (0.478)	−0.376 (0.436)
Proximity to ocean	−0.641 (0.771)	–	−0.694 (0.718)	–	0.572 (0.772)	–
Perceived coral health	0.233 (0.226)	–	0.067 (0.203)	–	−0.426* **(0.220)**	−0.438* **(0.206)**
Industry: recreational subsurface	0.790 (0.643)	–	0.652 (0.577)	–	−0.169 (0.588)	–
Industry: recreational surface	−0.513 (0.515)	–	−0.665 (0.490)	–	−0.377 (0.506)	–
Annual revenue (size)	−0.353 (0.212)	−0.296 (0.185)	−0.293 (0.203)	–	−0.180 (0.214)	–
Seniority (years with business)	−0.225 (0.169)	−0.219 (0.114)	-0.274 (0.158)	−0.211 (0.112)	−0.426* **(0.173)**	−0.391** **(0.128)**
Material/financial motivation (perceived value of coral reef services)	0.072 (0.062)	–	0.031 (0.055)	–	0.027 (0.056)	–
Material/financial motivation (perceived need for intervention)	−0.065 (0.128)	–	−0.052 (0.115)	–	−0.249* **(0.123)**	−0.240* **(0.113)**
Intrinsic (pro-nature) motivation	0.251** **(0.098)**	0.209** **(0.080)**	0.229** **(0.092)**	0.224** **(0.079)**	0.270** **(0.098)**	0.268** **(0.093)**
Reputation/social approval	−0.189 (0.165)	–	−0.049 (0.144)	–	0.284 (0.155)	0.280* **(0.138)**
	All variables	Best fit	All variables	Best fit	All variables	Best fit
AIC	214.62	197.59	236.43	219.13	222.85	205.14
Null deviance	208.40	208.40	233.39	233.39	234.94	234.94
Residual deviance	168.62	181.59	190.43	201.13	176.85	181.14
Hosmer–Lemeshow test	–	0.913	–	0.752	–	0.493

*p < 0.05.

**p < 0.01.

For the highest payment threshold (0.5% of annual revenue), ‘reputation/social approval’ was a significant and positive predictor of participation in coral insurance (*β* = 0.3 ± 0.1, *p* < 0.05, odds ratio of 1.3). In addition, at this highest threshold, ‘perceived coral health’, ‘perceived need for intervention’ (perception that reef health would not naturally restore itself), and ‘seniority’ (respondent’s years with the company) were negatively and significantly related to participation in coral insurance (Supplementary Fig. S2a; odds ratios of, respectively, 0.6, 0.8, and 0.7).

In addition to the models above, we analyzed ‘revealed preference’ (Supplementary Table S4), i.e., whether businesses had donated funds or staff time to coral conservation in the past. While it was not possible to verify past donations (third-party data did not exist), we label self-reported behavior as “revealed preference” because behavior was not hypothetical. Only two variables were statistically significant in this model: island and reputation/social approval. Reputation/social approval was significantly and positively correlated with past donations (*β* = 0.3 ± 0.1, *p* < 0.05, odds ratio of 1.3). In addition, businesses on Oʻahu were less likely to have donated to coral conservation in the past (*β* = -0.9 ± 0.4, *p* < 0.05, odds ratio of 0.4). All four models had Hosmer–Lemeshow values of *p* > 0.05 indicating no significant evidence of poor fit (Supplementary Table S4)**.**

## Discussion

Our study is the first to directly measure the business sector’s perception of coral health and value, and interest in financing coral restoration. Private-sector funding is necessary to achieve global biodiversity targets^9^, yet there remains a notable gap in the engagement of the private sector in conservation initiatives. We help to address this gap by providing critical new insight into private-sector buying potential for innovative solutions to conserve and restore coastlines, like nature-based insurance.

Overall, businesses rated coral value as low relative to widely used datasets quantifying ecosystem services^8,11^. In addition, business perceptions of coral health and value do not spatially align with coral cover or value metrics from, respectively, remote sensing and ecosystem services datasets^8,11,27^. In other words, regions where coral cover and ecosystem services were highest/lowest did not show any significant differences in businesses ratings of coral health and value.

Customer satisfaction, according to businesses, is decoupled from coral cover. This viewpoint was captured by one surf-shop manager who stated, “You can surf on a dead reef.” The majority of businesses reported very high customer satisfaction with customer “experiences in or around the ocean” regardless of surrounding coral cover. Customer experience in the ocean was not ranked as lower in heavily degraded coral reefs, even by dive and snorkel businesses that worked directly on the reef. Thus, in practice, businesses may see few financial incentives to help remediate reefs since, in their view, visitors will pay for on-reef experiences regardless of reef quality. This is consistent with many previous studies on ‘shifting baselines’ which demonstrate, for example, that divers are highly satisfied with ocean experiences despite ongoing reef degradation^29^. In addition, our results suggest that businesses generally overestimate the condition of corals where they operate, even when businesses work on the reef regularly. Business perceptions of coral cover were attuned to relative coral health within each island, as their ratings (Likert 1–5) closely matched intra-island quintiles of remotely sensed coral cover. However, remotely sensed coral cover was in a low quintile in many places perceived as “Fair” or “Healthy” when the former was rescaled based on a historic baseline.

Subsurface recreation (dive, snorkel, and spearfishing) businesses rated corals as significantly more valuable compared to other industries. In other words, businesses that operated on the reef saw a greater connection between coral reef health and business wellbeing. However, this pattern reversed for businesses operating on the ocean surface (kayak, surf, boating), which had one of the lowest coral value perceptions of any industry, including lodging and restaurants. Surface-recreation ratings of coral value were far lower than estimates from our ecosystem services dataset. Given that surface conditions depend heavily on reef morphology, this result was unusual and showed a pervasive lack of awareness of the importance of coral reefs to ocean states.

In terms of coral value to flood protection, around half of businesses surveyed were vulnerable to increased flooding if the topmost meter of coral reefs is lost in the future based on floodplain models^8^. Nonetheless, high-risk businesses were largely unaware that coral reefs offer them flood protection at present. Drivers of flood risk perception are multifaceted; however, several studies indicate that distance from the coast is a strong predictor of risk perception^30–32^. Since the majority (60.3%) of coastal businesses in Hawaiʻi are close to the ocean but not directly beachside (Supplementary Table S3), respondents may overestimate the protection afforded by slight coastal setbacks. Education on flood zone extents, sea-level rise, and the role of coral reefs in mitigating flood waters is necessary to bridge this disconnect.

Despite the low perception of coral reefs’ risk reduction values, most businesses opted into participating (binary yes/no variable) in coral restoration via insurance in our study sample. 68.7% of respondents affirmed that they would be interested in investing in coral insurance as described in Supplementary Fig. S3, with 43.6% of respondents assenting to the highest payment threshold of 0.5% annual revenue. Note that this study presented respondents with three discrete participation thresholds that are reasonably affordable yet sufficient to help sustain coral restoration (see Methods).

Business participation in coral restoration was removed from perceived material value of ecosystem services (Supplementary Table S5). Instead, the most consistent predictor of insurance buy-in was a preliminary “intrinsic (pro-nature) motivation” index (Fig. 5), which was highly significant and positively related to participation at all payment thresholds. ‘Intrinsic motivation’ is defined as pro-environmental behaviors driven by the inherent worthiness of nature rather than economic benefit; however, a more extensive survey is required to fully characterize intrinsic motivations. We presented oppositional statements such as, “Economic growth should be given priority in Hawaiʻi, even if the environment suffers to some extent” (inverse Likert scale), which we used as an early indication that business motivations diverged from coral economic use. However, intrinsic motivations may be present in respondents who do not view the environment v. economy as a dichotomy and therefore more neutral and extensive questions are required to fully explore intrinsic or non-use drivers of business decisions. Previous surveys of the general public^33^, including surveys in Hawaiʻi^34^, have highlighted non-use/non-financial values associated with coral reef conservation (e.g., bequest value, the desire to preserve reefs for future generations, and existence values, or the benefit of simply knowing that reefs exist). There is also evidence that norms and intrinsic motivation can drive ecotourism participation in private firms^35,36^. However, most prior research on non-monetary motivations in conservation has focused on individuals, e.g.,^37,38^ and not private firms. Our results point to the salience of non-use reef values in Hawaiʻi, but further research is needed to refine our understanding of these values in the tourism sector, e.g., whether a “warm glow” effect^39^ (participating in reef restoration for the joy of participation) is present in business actions.

One social variable, “business reputation/social approval,” was also positively correlated with participation in coral restoration through insurance at the highest threshold of 0.5% annual revenue and with “revealed preference” (past donations to reef protection). ‘Revealed preference’ may provide the most realistic gauge of participation because it is based on past behavior, rather than a hypothetical scenario. If businesses strongly agreed with the statements, “People in my community expect my business to help protect coral reefs” and “taking care of nature is my kuleana” (Hawaiian word for reciprocal relationship to my community), they were more likely to engage in past and potential reef spending. This finding again points to the need for further research to measure variants of ‘reputation’ or ‘social approval’ that are most salient for coral tourism, e.g., the role of social and internalized norms of behavior^40^, corporate image, or customer needs and wants (market risk)^23^ as drivers of business decisions. Several respondents mentioned that their customer base had shifted towards “green tourists” who demand eco-friendly business practices. Such ‘green tourism’ has been shown to motivate business decisions in the tourism industry^41^, and may likewise incentivize businesses in Hawaiʻi to fund reef restoration.

Indigenous Hawaiian ethnicity was significantly and positively linked to participation in coral restoration through insurance (Supplementary Fig. S2b). Native Hawaiian heritage may reflect place-based, indigenous ties to the land and ocean, and experiential knowledge of ocean risk and change^42,43^. Given the strong cultural and generational connection between Native Hawaiians and the ocean, it is unsurprising that these respondents were most willing to participate in coral restoration. However, coral restoration initiatives must avoid asking Native Hawaiian businesses to pay for environmental impact that has been created or exacerbated by colonization. Instead, Hawaiian business owners and community members should be supported in positions of restoration leadership. During the survey, many respondents noted a fear of government mismanagement of private sector funds, and several respondents noted, “We would only participate if [a community nonprofit] was in control”. Future coral restoration efforts must be planned carefully to cultivate trust and prioritize local leadership.

Respondents who were not willing to participate listed several reasons but lack of trust in centralized organizations and fear of corruption was the main deterrent. Businesses also emphasized that public education was a higher priority than a coral restoration/insurance fund, while noting that tourists often “can’t tell the difference between a coral and a rock.” Other businesses cited financial difficulties due to closures during the COVID-19 pandemic. In addition, many respondents stated that large developers, large agribusiness, and tourists who harm the reef^10^ should pay for coral restoration. Finally, at least one respondent noted a lack of individual efficacy, i.e., that environmental problems in Hawaiʻi are too large and global to tackle, and individual firms have little control over reef evolutionary trajectories. Notably, many businesses mentioned that they supported specific reef regulations like bans on toxic sunscreen and temporary closures of Hanauma Bay, highlighting business support for efforts to limit tourism impacts, even at the expense of businesses themselves.

Our results highlight several factors that can help reef financing programs succeed. Given the importance of reputational motivations, improving visitor education, e.g., through on-flight advertisements or sustainability pledges, may help bolster green customer behavior that motivates business decisions. Furthermore, our results underscore a disconnect between the tangible benefits provided by coral reefs and awareness of these benefits among private businesses. Education initiatives are needed to communicate the economic benefits of coral reefs and overcome the disconnect between perceived and calculated value of coral reefs. High transparency, democratic decision-making, and leadership by well-known and trusted community groups may address the lack of trust voiced by many survey respondents. Local leadership is particularly important to steer and allocate private funding for coral restoration, as large multinational entities can garner substantial donations but are detached from to local priorities. Finally, coral restoration programs can be designed to welcome hands-on participation by businesses and their customers; for example, businesses can lend labor and facilities to reef repair. Recent literature has demonstrated that hands-on reef restoration is popular among the general public^33^, and coral insurance may be a vehicle for scaling up the tourist sector’s involvement in restoration^44^.

Our survey was limited in that it addressed relatively few large multinational companies like major hotel chains, where decision makers are harder to access by survey. In the future, interview- or focus-group research on multinational companies could help fill this gap. Further research is needed to 1) understand incentives for large firms to pay for reef restoration and 2) draft equitable financing structures that place a proportional burden on wealthy companies while maintaining community and especially indigenous leadership. Coral reef insurance and similar initiatives, if planned democratically, may help generate local employment opportunities in reef restoration and reduce the leakage of tourism revenue outside the islands. Furthermore, the Social-Ecological Systems (SES) framework may be used in future research towards optimizing coral insurance design and siting^36^. For example, the SES framework indicates that successful voluntary initiatives may be affected by the size of the resource system^45^. Would focusing coral reef insurance on a limited region help generate peer expectations to participate? SES analyses indicate that system productivity (e.g., reef condition) has a curvilinear relationship with voluntary firm participation in conservation^36^. What perceived reef state is too degraded (or not degraded enough) to drive participation? Finally, one limitation of our study was that possible biases such as “yea-saying” (acquiescence) could not be completely avoided, i.e., respondents may have replied falsely in order to appear generous or avoid decision tradeoffs. Future research could incorporate additional correctives to acquiescence, such as offering multiple, opposite-keyed indicators of a construct, e.g., asking questions about both willingness and specific reluctance to participate^46^. We could also improve the reliability of results by itemizing levels of uncertainty associated with each reef restoration approach funded by coral insurance^47^. While we were limited by time available from businesses and population size (a limited number of coastal businesses exist on small islands), future versions of this work could address a larger geographic area, thus allowing for a higher sample size and random rather than purposive sampling, and include additional survey questions to test further for biases.

This study addresses coral ecosystem services from the perspective of coastal businesses that can be key beneficiaries and decision makers of coastal adaptation needs and public/private investment. While coral ecosystem services are increasingly well quantified, these services must be better communicated to beneficiaries (coastal businesses), who often do not view reefs as economically valuable. In addition, this study further provides early evidence of the importance of reputation, identity, and regard for nature as inherently worthy of protection, in driving payments for conservation and restoration. These levers may motivate a new community of businesses that have strong interest in protecting the coastline and cultivating green tourism.

## Methods

We conducted our research on two Hawaiian islands: Hawaiʻi Island and Oʻahu. Coral reefs in Hawaiʻi are threatened by marine heatwaves and hurricanes, yet coral reefs in Hawaiʻi generate net economic benefits of $360 million per year^48^ and many highly developed coastlines in Hawaiʻi receive annual benefits of $10 million km^−1^;^8^. Based on flood risk information from Reguero et al. 2021^8^ in 2020, The Nature Conservancy (TNC) conducted a feasibility assessment of parametric coral insurance in Hawaiʻi, determining that coral insurance would be feasible in the State^18^. We selected Oʻahu and Hawaiʻi for our study sites because these islands represent opposite ends of the development spectrum. Oʻahu has the highest number of visitors and extremely high property values that bolster the economic value of coral reefs to tourism and flood protection; however, coral reefs on Oʻahu are highly degraded. Hawaiʻi Island is comparatively undeveloped with higher coral cover, but its economy is growing. These islands therefore contain businesses across a wide spectrum of coral reef health, tourism, and storm pressure, maximizing variability in our sample.

We surveyed 202 coastal businesses in the tourism industry, which operated at 259 locations. Surveys were conducted between August 2021 and May 2022. 156 surveys were conducted in person and 46 surveys online, using an identical protocol for each method. Online surveys were administered via email using Qualtrics™ survey management software (response rate of 19% via email and 87% in person). Given that our survey population was limited in size, we used purposive sampling to identify businesses that fell under the criteria above and approached all businesses that were discoverable in person or online. We excluded businesses that did not have a permanent facility or storefront, such as mobile dive operators and food trucks, given that our study required permanent location information. To ensure consistency in data collection, all in-person surveys were conducted by a single individual (R. R. Carlson).

We developed our survey protocol through a literature review, interviews with experts in behavioral economics, and a pretest of our survey with 12 coastal business owners and operators in Maui, whose responses were not included in results. Pretesting and interviews helped to refine survey language so that questions were easy to understand and effectively operationalized covariates. Supplementary Table S5 itemizes a full list of variables we analyzed and associated survey questions. The survey covered several possible motivations for funding restoration. These motivations included the perceived, material value of corals to business revenue and operations. They also included alternative motivations called “other-regarding preferences” in behavioral economics literature: reputation/social approval and intrinsic motivation to protect coral as worthy of protection in itself (rather than an economic asset)^28^. These questions were designed to provide early indications of non-monetary motivations in business decisions, but a full exploration of diverse types of other-regarding preferences salient in this tourism community was outside the scope of this study and is an area identified for further research (see Discussion). Questions also addressed structural features of businesses (e.g., age of business, whether businesses were located only in Hawaiʻi or multinational) and traits of the individual respondent (e.g., age, years with company). Finally, the survey presented a payment scenario surrounding coral reef insurance, described below (See “Payment for coral insurance”). The interview protocol was approved by Stanford University’s Institutional Review Board for human subjects research on June 6, 2021, protocol #61,641 and all participants provided informed consent. All experiments were performed in accordance with relevant guidelines and regulations.

### Perception of coral health and value

The first goals of our study were to assess 1) business perception of coral health and value and 2) differences in perception across varying industries, coral cover, and ecosystem service levels, as mapped using external datasets. For example, in areas where corals provide high ecosystem services (e.g., flood protection), do businesses value coral reefs highly? Which industries see strong financial interest in coral reefs?

To address these questions, we first asked businesses a series of questions related to coral health and value, with responses rated on a 5-point Likert scale (Supplementary Table S1). We then compared perceived coral health (survey response) to remotely sensed coral cover. Likewise, we compared perceived coral value (survey response) to two geospatial datasets on coral reef ecosystem services: coral value to tourism and recreation ($ ha^-1^) and coral value to flood protection (Supplementary Table S1). We evaluated whether business perception of reef health and value differed across industries, remotely sensed coral cover, and ecosystem service levels.

#### Coral health

We determined perceived coral health from the question, “How would you characterize the reef closest to where you conduct business?” where businesses responded on a Likert scale of 1–5 (“Very unhealthy” to “Very healthy”). We then determined percent coral cover surrounding each business from a dataset by the Global Airborne Observatory (GAO), an aircraft that mapped coral cover to 16 m depth across the main Hawaiian Islands at 2 m spatial resolution using Airborne Imaging Spectroscopy, a form of extremely high-resolution remote sensing. GAO data have been validated in the field with R^2^ = 0.94^27^.

To evaluate coral cover near business locations, we found the closest shoreline location to each business, then determined coral cover as the mean and maximum GAO pixel value of coral cover within a 1 km buffer around that location. If businesses were located next to a harbor, we evaluated coral cover around the closest shoreline location outside of the harbor given that most corals exist outside of the built environment. For marine operators (e.g., snorkel charters), we asked respondents to list the top three ocean locations they visit and evaluated mean and maximum coral cover within 200 m of those points. Marine operators typically target mooring buoys and operate within a small radius of their target location, so 200 m was sufficient to gauge coral cover at ocean sites.

Remotely sensed coral cover needed to be rescaled, since survey questions used a 5-point Likert scale and GAO data was continuous (1–100% live coral cover). We converted GAO maps to a 1–5 scale using two separate methods:

- All pixels were divided into intra-island quintiles (1–5) of percent coral cover, and corals within the stated buffer distance of businesses were graded based on mean quintile.
- Coral cover values 1–5 were assigned to GAO pixels based on a literature review of coral cover in Hawaiʻi since 1999^49^. Mean historic cover from 1999–2008 was ~ 20%, so we assigned a value of 3 to each pixel with 15–25% coral cover and ranked lower/higher coral cover as 1–5 accordingly.

To compare remotely sensed and perceived values, we identified businesses in the lowest and highest percentiles of GAO coral cover, i.e., businesses with < 1% (low) and 10–24% (high) mean GAO coral cover. We then used bootstrapping to compare mean business rating of coral health between these areas. To bootstrap, we sampled with replacement from low and high GAO coral cover groups with 10,000 iterations to estimate 95% Confidence Intervals (CIs) of survey responses (perceived coral health) from each group. Non-overlapping 95% CIs indicated significant differences between groups. Next, for each business in our dataset, we calculated a “closeness index” as the business rating of coral health (survey response) minus rescaled (1–5) GAO coral cover. The “closeness index” captures the spatial match/mismatch between business estimates and remote-sensing data and is not intended as an index of business perception “accuracy,” since the Likert scale is not quantitatively comparable to continuous coral cover. We used bootstrapping to compare the “closeness index” between islands and industries, i.e., dive and snorkel recreation, watersports, retail, restaurants and hotels. This allowed us to estimate whether certain groups of businesses over- and under-estimated coral health relative to remote sensing.

We also assessed whether customer experience in the ocean differed significantly between low and high GAO coral cover areas. We asked businesses to respond to the statement, “My customers are generally satisfied by their experience in or around the ocean” (5-point Likert scale) and used bootstrapping to compare means between the lowest and highest GAO coral cover areas.

#### Coral value

To evaluate coral value at business locations, we considered two types of value: coral value to recreation/tourism and coral value to flood protection. We determined business-perceived coral value to recreation/tourism from the mean response to two survey questions: “I am worried that declining coral reefs will affect my business” and “If more of the reef died, I would have fewer customers” (“Strongly disagree” to “Strongly agree” on the Likert scale 1–5; Supplementary Table S1). We then used an external ecosystem services dataset, MOW^11^, to map the recreational/tourism value of coral reefs at 1 km spatial resolution in Hawaiʻi. This dataset, described fully in Spalding et al.^11^, was based on 1) density of social media photos on coral reefs arising from each location, 2) dive shop abundance, and 3) a set fraction of tourist spending in coral-adjacent areas. We overlaid the MOW geospatial dataset with business operating locations and found recreational value at those locations. As with GAO data, we rescaled MOW data into ordinal quintiles (1–5) to facilitate comparison with survey questions.

Finally, we determined perceived coral value to flood protection from business response to the statement, “If more of the reef died, physical assets of my business could be damaged” (1–5 Likert scale from “Strongly disagree” to “Strongly agree”). We used floodplain projections from Reguero et al.^8^ that provide flood zones at present and with coral reef degradation. The differences between both scenarios were used to quantify economic benefit of the risk reduction provided by coral reefs in terms of flood protection. The results were calculated in 100 m sections of coastlines. We mapped floodplains and flood depths against business locations and determined whether each business can anticipate deeper flooding during a 10-year flood if 1 m of corals are lost (binary variable, Yes/No). We elected to use 10-year floodplains because these flood levels are lower (more conservative) than 50- or 100-year floodplains, and because certain businesses may use short time horizons for risk assessment. In addition, coral reef flood protection values are relative constant in relative terms across different return periods^8^. Note that we used economic values in direct flood protection as our focus was on private sector interest, but other metrics related to social vulnerability may be also relevant for community decision making.

To compare business perception with ecosystem services mapping, we used the same procedure as with coral health. We used bootstrapping to compare mean perceived coral value between businesses located in areas with high and low reef value based on MOW. We also used bootstrapping to compare business rankings of coral reef value in areas with and without flood protection from coral reefs based on Reguero et al.^8^. As above, we compared the mean ‘closeness index’ between islands and industries, using the ‘closeness index’ to visualize spatial match/mismatch between business-perceived value and MOW while understanding that the two indices are not quantitatively comparable.

Finally, we visually compared perceived coral health and value to external datasets by reprojecting all survey data in Supplementary Table S1 on a hexagonal grid (2 km edge length), which provided a consistent mesh for comparing overlapping spatial data while also generalizing sensitive business locations, as advised by previous studies^50^.

Several companies had multiple locations in Hawaiʻi, and/or represented multinational entities. For companies with multiple locations inside Hawaiʻi, we evaluated the average coral cover and value of all locations only if the interviewee reported “full control” over budgeting and financial decisions at the company level. This level of authority implied oversight over company-wide activities that were not limited to a single facility. For multinational entities, we did not evaluate locations outside of Hawaiʻi because coral data were unavailable elsewhere.

### Payment for coral insurance

To estimate business payment for coral reef insurance, we used a variant of a dichotomous choice stated preference survey, which is widely used in environmental economics to quantify Willingness to Pay (WTP) for natural resources and ecosystem services. We adapted discrete choice questions used in previous surveys to estimate WTP for coral conservation^51–53^ with adjustments for our use case. Notably, our analysis departs from traditional WTP studies^54^ in that we did not evaluate a mean or median WTP but instead analyzed business participation (binary yes/no) in an insurance program (described in Fig. S3 and adapted from previous coral insurance pilots^18–21^) with a stable buy-in threshold (see “Model Specification” below). For clarity, we therefore use the terms “participation” or “buy-in” rather than WTP throughout this text. We first asked respondents if they would pay a specific price (referred to in WTP protocols as a “bid”) for a natural resource, with a binary choice of “Yes” or “No”. We used a double-bounded dichotomous choice survey approach, in which a “Yes” response prompted a follow-up question asking if the respondent would pay a higher bid. Conversely, if the respondent answered “No” to the first bid, they were asked if they would pay a lower bid. We opted for this method because it allowed us to gather feedback on several bid values while reducing the cognitive burden of the survey (only Yes/No responses required). This efficiency was critical in our study as most respondents were only willing to participate in a survey under ~ 15 min long.

We anticipated several biases that we accounted for in survey design. These include shift effects, in which follow-up questions alter respondents’ conception of the value of the good itself. For example, if a respondent answers “Yes” to the initial bid, a higher, follow-up bid may be seen as a gratuitous attempt by the surveyor to raise revenue. In addition, dichotomous choice is prone to “yea-saying,” in which respondents claim high WTP regardless of their actual intention to pay^51^. This is particularly common when payment appears to be the “right” or ethical choice, as with environmental causes. “Yea-saying” is prevalent when surveys are conducted in person, as respondents may aim to please the surveyor. As one respondent in our survey noted, “I don’t want you to think I’m a bad person.”

To reduce these biases, we stressed surveyor neutrality and emphasized the fact that the coral insurance scenario was, at the time of the survey, actively under planning for the State of Hawaiʻi (it has since been implemented), so that respondents considered their serious intent to pay. In addition, we did not include any respondents in the analysis who reported “no control” over budgetary decisions at the company level; we further included “seniority” (collinear with budgetary control; see Model Specification) in the analysis and therefore controlled for varying levels of budgetary authority. To account for “yea-saying,” we included a question to elicit “revealed preferences,” i.e., participation as revealed by past behavior. We asked respondents to state whether they had donated to ocean-related community groups or paid for staff time in ocean conservation (e.g., beach cleanups) in the past year, and to name specific details. Past investment in coral reefs may signal realistic participation (“what respondents do, rather than what they say”)^55^, and we therefore considered this “revealed” behavior as an alternative dependent variable. We rated “revealed payment” as 1 if respondents had donated either money or paid staff time to reef conservation in the past year, and 0 if not. Finally, we prevented selection bias (e.g., more frequent survey participation by already-interested firms) by conducting the majority of surveys in person, where survey response rate was high (87%).

### Model specification

Our analysis represented a departure from traditional WTP estimation methods. For example, the Contingent Valuation WTP method requires that the initial bid vary randomly among survey participants, and consequent lower/higher secondary bids also vary, so as to estimate a utility function and discrete mean and median WTP for the survey population^56^. Our experiment, in contrast, did not seek to identify a discrete WTP, or determine the maximum payment possible from businesses. Rather, we worked with conservation partners (TNC) who were actively designing a coral insurance program; we sought to estimate the likelihood of businesses opting into/out of (binary outcome) three optional payment thresholds that were determined a priori as relevant in a realistic insurance program. As such, our modeling approach (see below) is derived from Grillos^57^, who estimated payments for ecosystem services in an active NGO-implemented program, rather than theoretical WTP models. We presented respondents with the same starting bid, which was deemed realistic within the context of the Hawaiʻi coral insurance pilot. Given that we surveyed businesses of varying sizes, our bids were portions of revenue (e.g., 1/4 of 1% of annual revenue) rather than a flat fee. Bids are outlined in (Supplementary Table S2).

Following Grillos^57^, and given that our dependent variable was binary (1 = “Yes” to bid, 0 = “No” to bid), we modeled participation in coral insurance using logistic regression. While probit models are also possible in dichotomous choice analysis, odds ratios given by logit models have a linear relationship to explanatory variables, thus making effect sizes easier to interpret. Our data was grouped into two islands, Oʻahu and Hawaiʻi Island, and we therefore used “island” as a fixed effect. Random effects were not appropriate as the number of groups (two islands) was small, and because initial mixed-effects models converged on little to no evidence of non-independence within the “island” group^58^.

We defined $p({X}_{ij})$ as the probability of a business responding “Yes” to a bid, where ${X}_{ij}$ is a matrix of covariates associated with participant *i* on island *j*. Thus, the probability that a business will pay for coral insurance was modeled as:

1 $$log\left(\frac{p({X}_{ij})}{1 - p({X}_{ij})}\right)={\beta }_{0}+\beta ({X}_{ij})$$

where ${\beta }_{0}$ is the model intercept and $\beta$ is a sequence of regression coefficients. Covariates ${X}_{ij}$ modeled for each participant are listed in Supplementary Table S5. We checked covariates for correlation using the following methods: Pearson’s correlation between ordinal variables (all correlations *r* < 0.7); Point Biserial correlation between categorical and ordinal variables; and chi-squared test between categorical variables. The variables “seniority” and “influence” were correlated and we therefore opted to use “seniority” (years with the company) as a proxy for “influence” (level of control over company budget).

As was stated previously, the goal of this study was to assess drivers of business participation at three static buy-in thresholds. We thus applied Eq. (1) to three models corresponding to our three payment thresholds for a coral insurance program with n = 171 (excluding 31 businesses who were missing at least one covariate value listed in Table 1).

- Model 1: Participation in coral insurance at ¼ of 1% of annual revenue.
- Model 2: Participation in coral insurance at ½ of 1% of annual revenue.
- Model 3: Participation in coral insurance at $${\raise.5ex\hbox{$\scriptstyle 1$}\kern-.1em/ \kern-.15em\lower.25ex\hbox{$\scriptstyle 8$} }$$ of 1% of annual revenue.

We also modeled “revealed” payment, a binary variable, using the same logistic regression approach.

- Model 4: Past participation in coral restoration and/or conservation through donations or paid staff time

We selected all models using a best-subset selection procedure^59^, in which we examined all possible combinations of covariates to select the model of best fit based on deviance and AIC values. We used the following procedure:

An initial model (M_0_) contained no predictors (i.e., sample mean). For k = 1, 2,…, p:

- We fit $(\frac{p}{k})$ models comprised of *k* predictors.
- We picked M_k_ as the model of best fit from $(\frac{p}{k})$ models, where best fit was determined based on the lowest deviance score, analogous to the Residual Sum of Squares (RSS) in ordinary least squares regression.

We selected a single best model from M_0_,…,M_p_ using the AIC value which, unlike deviance, does not necessarily decrease as *k* increases.

Finally, we used a Hosmer–Lemeshow test to determine whether any final models showed evidence of poor fit, which was not the case. Model fitting and validation was completed using the *bestglm* and *ResourceSelection* packages in R^60,61^.

## Supplementary Information

Supplementary Information 1.

## Data Availability

All code supporting this analysis is available at https://www.github.com/rrcarlson/coral_insurance. Survey data cannot be published to protect the privacy of survey participants. Remote sensing data on coral cover are available at Zenodo (https://zenodo.org/record/4777345 based on Asner et al.^27^). Data on coral value are available at the Mapping Ocean Wealth dataset (coral value to recreation and tourism; https://maps.oceanwealth.org/) and ScienceBase (coral value to flood protection; https://doi.org/10.5066/P9KMH2VX based on Reguero et al.^8^).
