# Supplementary material for: Coastal business perception of coral value and payment for coral restoration
Source: Sci Rep. 2025 Mar 18;15:9285. doi: 10.1038/s41598-025-93439-x (PMC11920439; doi:10.1038/s41598-025-93439-x)
Supplement: Supplementary file 1 — Supplementary Information 1. [file 41598_2025_93439_MOESM1_ESM.docx]

**Supplementary Information for**

# Coastal business perception of coral value and payment for coral restoration

Rachel R. Carlson^1,3,4,9^, Joanna Klitzke^1,2^, Gretchen C. Daily^5,6,7^, Larry B. Crowder^4,5,7^, Borja G. Reguero^8^, Gregory P. Asner^3^

^1^ Emmett Interdisciplinary Program in Environment and Resources, Stanford University, Stanford, CA, United States

^2^ Stanford Graduate School of Business, Stanford University, Stanford, CA, United States

^3^ Center for Global Discovery and Conservation Science, Arizona State University, Hilo, HI, United States

^4^ Hopkins Marine Station, Stanford University, Monterey, CA, United States

^5^ Department of Biology, Stanford University, Stanford, CA, United States

^6^ Natural Capital Project, Stanford University, Stanford, CA, United States

^7^ Woods Institute for the Environment, Stanford University, Stanford, CA, United States

^8^ Coastal Science and Policy, University of California, Santa Cruz, Santa Cruz, CA, United States

^9^ Department of Environmental Science, Policy, and Management, University of California, Berkeley, Berkeley, CA, United States

**Note: Citations refer to References in the main text.**

**Table S1:** Coral health and value metrics comparing business perception (survey indicators) with external datasets.

| **Variable** | **Business perception** | **Actual condition** |
| --- | --- | --- |
| Coral health | How would you characterize the reef closest to where you conduct business?*  *(Very Unhealthy to Very Healthy, 5-point scale)*  *Ocean operators were asked this question for each of top three ocean locations and we derived the mean and maximum of the three responses. | Mean and maximum % live coral cover within   - 1 km of land-based business location or - 200 m of top three ocean operating locations.   Based on Global Airborne Observatory coral cover, 2 m^2^ spatial resolution^27^. |
| Coral value to tourism and recreation | Mean value of two responses:   - I am worried that declining coral reefs will affect my business *(Strongly Disagree to Strongly Agree, 5-point scale)* - If more of the reef died, I would have fewer customers *(Strongly Disagree to Strongly Agree, 5-point scale)* | Mean tourist and recreation value of coral reefs (USD ha^-1^ year^-1^) within   - 1 km of land-based business location or - 200 m of top three ocean operating locations.   Based on Mapping Ocean Wealth dataset, 1 km^2^ spatial resolution^11^ |
| Coral value to flood protection | If more of the reef died, physical assets of my business could be damaged *(Strongly Disagree to Strongly Agree, 5-point scale)* | Binary variable: Y/N business location will experience an increase in 10-year flood depth after 1 vertical meter of reef loss.  Based on Reguero et al.^8^ |

**Table S2:** Bid levels presented to survey participants.

| **Starting bid (constant)** | **Follow-up question if answered “Yes” to starting bid** | **Follow-up question if answered “No” to starting bid** |
| --- | --- | --- |
| ¼ of 1% of annual revenue | ½ of 1% of annual revenue | ⅛ of 1% of annual revenue |
| Bid levels were translated to a dollar value based on the stated annual revenue of each business. | | |

**Table S3**: Business survey response statistics for each independent and dependent variable.

| **Variables** | **Survey response** | ***Portion of responses*** |  |  |
| --- | --- | --- | --- | --- |
| **Demographics** |  |  |  |  |
| Distribution method | Email | *41 (21.1%)* |  |  |
|  | In person | *153 (78.9%)* |  |  |
| Years in Hawaiʻi | 0-5  5-10  10-15  15-20  Over 20 | *24 (12.4%)*  *24 (12.4%)*  *5 (2.6%)*  *34 (17.5%)*  *107 (55.2%)* |  |  |
| Identity/ethnicity | Hawaiian (indigenous)  “Born and raised” but not Hawaiian  Permanent resident  Seasonal or temporary resident  Other | *38 (19.6%)*  *25 (12.9%)*  *123 (63.4%)*  *3 (1.5%)*  *5 (2.6%)* |  |  |
| Age | 18 – 25  26 – 40  41 – 55  56 – 70  71 + | *38 (19.6%)*  *65 (33.5%)*  *60 (30.9%)*  *27 (13.9%)*  *4 (2.1%)* |  |  |
| Gender | Female  Male  Non-binary/other | *93 (47.9%)*  *98 (50.5%)*  *3 (1.5%)* |  |  |
| Years with business | < 5 years  5 – 10 years  11 – 15 years  16 – 20 years  > 20 years | *98 (51.3%)*  *30 (15.7%)*  *15 (7.9%)*  *12 (6.3%)*  *36 (18.8%)* |  |  |
| Influence on budget | Slight  Significant  Full control | *66 (34.6%)*  *68 (35.6%)*  *57 (29.8%)* |  |  |
| **Business structural features** |  |  |  |  |
|  |  |  |  |  |
| Island | Oʻahu  Hawaiʻi  Multiple islands | *137 (71.4%)*  *50 (26.0%)*  *5 (2.6%)* |  |  |
| Proximity to ocean | Beachfront  <1 mile (not beachfront)  Inland | *59 (30.4%)*  *117 (60.3%)*  *18 (9.3%)* |  |  |
| Coral health nearby | Scale of 1-5 | *μ = 2.42± 0.92* |  |  |
| Coral health 10 years ago | Scale of 1-5 | *μ = 3.44 ± 1.01* |  |  |
| Industry | Recreational surface  Recreational subsurface  Coastal retail, restaurant, or lodging | *42 (21.6%)*  *37 (19.1%)*  *115 (59.3%)* |  |  |
| Annual revenue | < $10k  $10k - $100k  $100k - $1M  $1M - $5M  $5M - $10M  $10M + | *3 (1.6%)*  *26 (14.3%)*  *87 (47.8%)*  *48 (26.4%)*  *9 (4.9%)*  *9 (4.9%)* |  |  |
| **Material + alternative motivations** |  |  |  |  |
|  |  |  |  |  |
| **Material motivations:**  Perceived value of coral services | Scale of 1-15 (see questions in Table S5) | *μ = 8.75 ± 3.66* |  |  |
| Perceived need for intervention | Scale of 1-10 (see questions in Table S5) | *μ = 6.94 ± 2.05* |  |  |
| **Other-regarding preferences:** Intrinsic (pro-nature) motivation | Scale of 1-15 (see questions in Table S5) | *μ = 12.65 ± 2.25* |  |  |
| Business reputation | Scale of 1-10 (see questions in Table S5) | *μ = 8.88 ± 1.39* |  |  |
| **Dependent variable** | | |  | Response variable |
|  |  |  |  |  |
| Willingness to pay (WTP) for coral reef insurance | *No WTP* | *56 (31.28%)* |  |  |
|  | *WTP at most 1/8 of 1% annual revenue* | *23 (12.85%)* |  |  |
|  | *WTP at most 1/4 of 1% annual revenue* | *22 (12.29%)* |  |  |
|  | *WTP at most 1/2 of 1% annual revenue* | *78 (43.58%)* |  |  |

**Table S4**: Drivers of willingness to pay as “revealed preference,” i.e., past donation of money or staff time to coral reef conservation. Regression coefficients ± standard deviation shown.

|  | **Revealed preference (past payment for coral conservation)** | |
| --- | --- | --- |
|  | All variables | Best fit |
| **Distribution method**  (Effect shown: in person) | -0.669  (0.582) | -- |
| **Years in Hawaiʻi** | -0.101  (0.181) | -- |
| **Identity/ethnicity**  (Effect shown: Hawaiian/indigenous) | 1.573  (0.886) | -- |
| **Age** | 0.224  (0.256) | 0.310  (0.184) |
| **Gender**  (Effect shown: Male) | 0.062  (0.429) | -- |
| **Island**  (Effect shown: Oʻahu) | **-1.097***  **(0.500)** | **-0.923***  **(0.403)** |
| **Proximity to ocean** | 0.787  (0.789) | -- |
| **Perceived coral health** | -0.057  (0.217) | -- |
| **Industry: recreational subsurface** | 0.319  (0.594) | -- |
| **Industry: recreational surface** | 1.112  (0.584) | -- |
| **Annual revenue (size)** | -0.089  (0.216) | -- |
| **Seniority (years with business)** | -0.081  (0.171) | -- |
| **Perceived value of coral reef services** | 0.017  (0.058) | -- |
| **Perceived need for intervention** | -0.049  (0.120) | -- |
| **Intrinsic (pro-nature) motivation** | 0.004  (0.096) | -- |
| **Reputation/social approval** | -0.232  (0.164) | **0.300***  **(0.133)** |

|  | **Revealed preference (past payment for coral conservation)** | |
| --- | --- | --- |
|  | All variables | Best fit |
| **AIC** | 209.78 | 189.5 |
| **Null deviance**  **Residual Deviance** | 195.14  163.78 | 195.14  179.50 |
| **Hosmer-Lemeshow test** | -- | 0.799 |

* *p* < 0.05

** *p* < 0.01

**Table S5:** Independent variables and survey indicators. Some variables were derived as the sum of responses to multiple questions; in these cases, the inter-item correlation is noted (acceptable values: 0.15 - 0.50).

| **Independent variable** | **Indicator on survey** |
| --- | --- |
| **Material motivations** | |
| Perceived value of coral reef services | I am worried that declining coral reefs will affect my business *(Strongly agree/Strongly disagree, 5-point scale)*  If more of the reef died, physical assets of my business could be damaged *(Strongly agree/Strongly disagree, 5-point scale)*  If more of the reef died, I would have fewer customers *(5-point scale)* |
| Perceived need for intervention  *(inter-item correlation: 0.20)* | Science and technology will eventually solve our problems with pollution, overpopulation, and diminishing resources *(inverse, Strongly agree/Strongly disagree, 5-point scale)*  If more of the reef died, it would probably come back naturally *(inverse, Strongly agree/Strongly disagree, 5-point scale)* |
| **Other-regarding preferences** | |
| Intrinsic (pro-nature) motivation  *(inter-item correlation: 0.15)* | It’s good that politicians and authorities try to make Hawaiian businesses act more environmentally friendly *(Strongly agree/Strongly disagree, 5-point scale)*  The reduction in tourism during COVID was good for Hawaiʻi *(Strongly agree/Strongly disagree, 5-point scale)*  Economic growth should be given priority in Hawaiʻi, even if the environment suffers to some extent. *(inverse, Strongly agree/Strongly disagree, 5-point scale)* |
| Reputation/social approval  *(inter-item correlation: 0.27)* | People in my community expect my business to help protect coral reefs *(Strongly agree/Strongly disagree, 5-point scale)*  Taking care of nature is my kuleana (Hawaiian for reciprocal relationship to my community) *(Strongly agree/Strongly disagree, 5-point scale)* |
| **Demographics** | |
| Distribution method | Email, in person |
| Years in Hawaiʻi | How long have you lived on island? |
| Identity/ethnicity | Do you identify as any of the following? *(list of identities/ethnicities)* |
| Age | How old are you? |
| Gender | What gender do you identify with? |
| Years with business | How many years have you been with this company? |
| Influence on budget | How much influence do you have over budgeting and financial decisions? |
| **Business structural features** | |
| Island | *(Hawaiʻi Island/Oʻahu)* |
| Proximity to ocean | Which of the following best describes your primary business location? *(Beachfront/Close to the beach/Inland/Other)* |
| Perceived coral health | How healthy is the reef in (listed business location)? |
| Industry | What type of business do you work for? |
| Annual revenue | What was your estimated gross annual revenue for 2019 (pre-pandemic)? This question helps us understand the size of your business. |
| Locations outside of Hawaiʻi | Do you have any business locations outside of Hawaiʻi? |

**
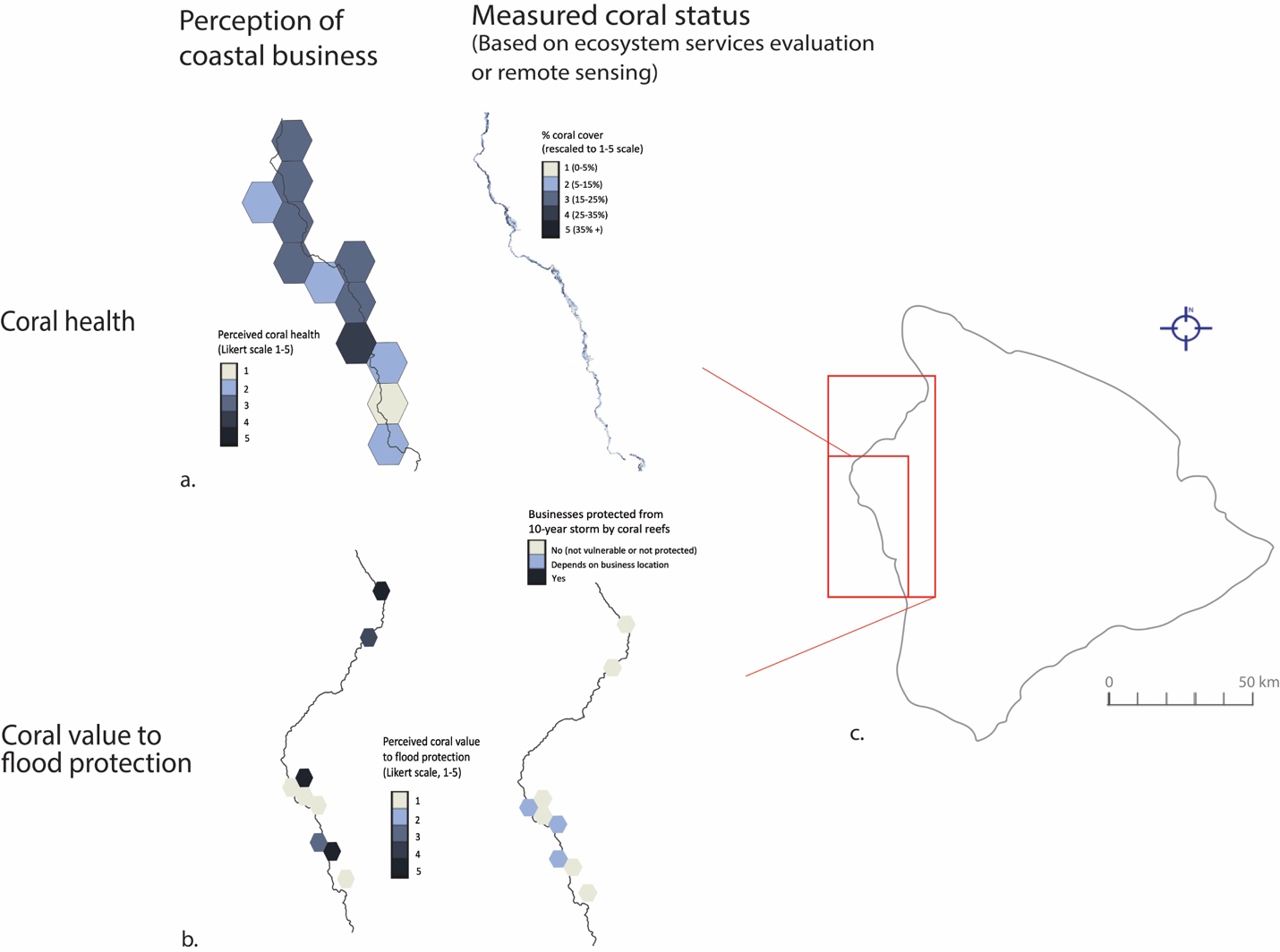
**

**Figure S1:** Map of Hawaiʻi Island showing: **a)** Business perception of coral health compared to remotely sensed coral cover, where both are compared on a 1-5 scale. **b)** Business perception of coral value to flood protection v. areas where corals confer flood protection (yes/no) in a 10-year storm^8^. Relative to flood projection data^8^, businesses overestimate coral value to flood protection in many regions of Hawaiʻi Island. **c)** Boundaries of a and b on Hawaiʻi Island.


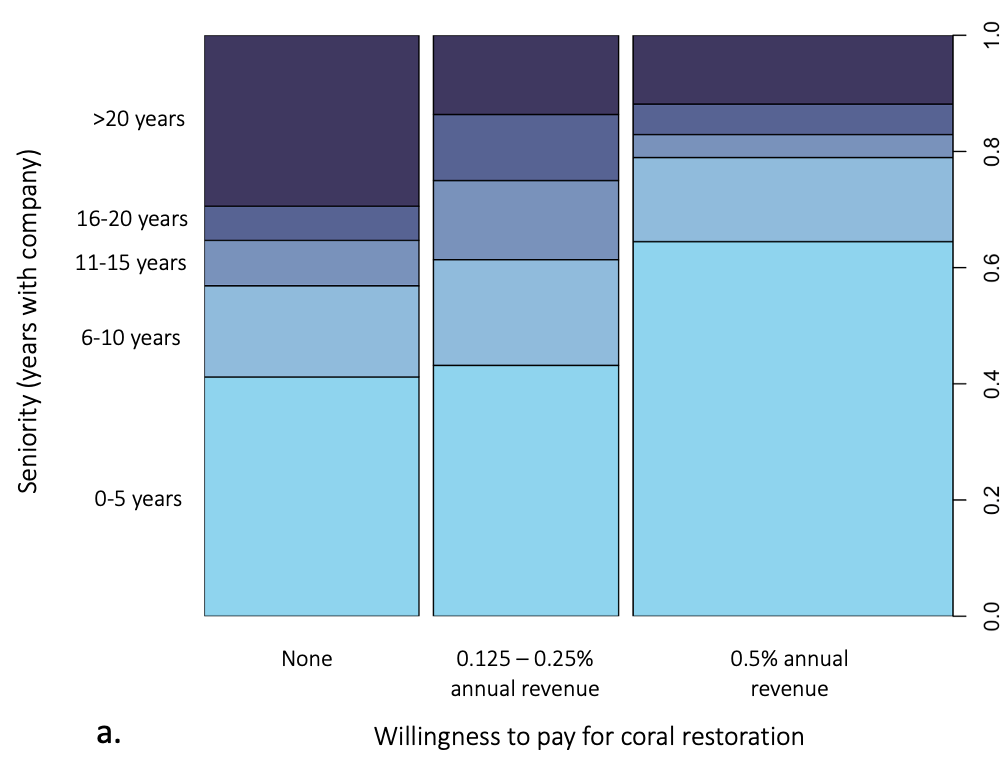


Share of total responses


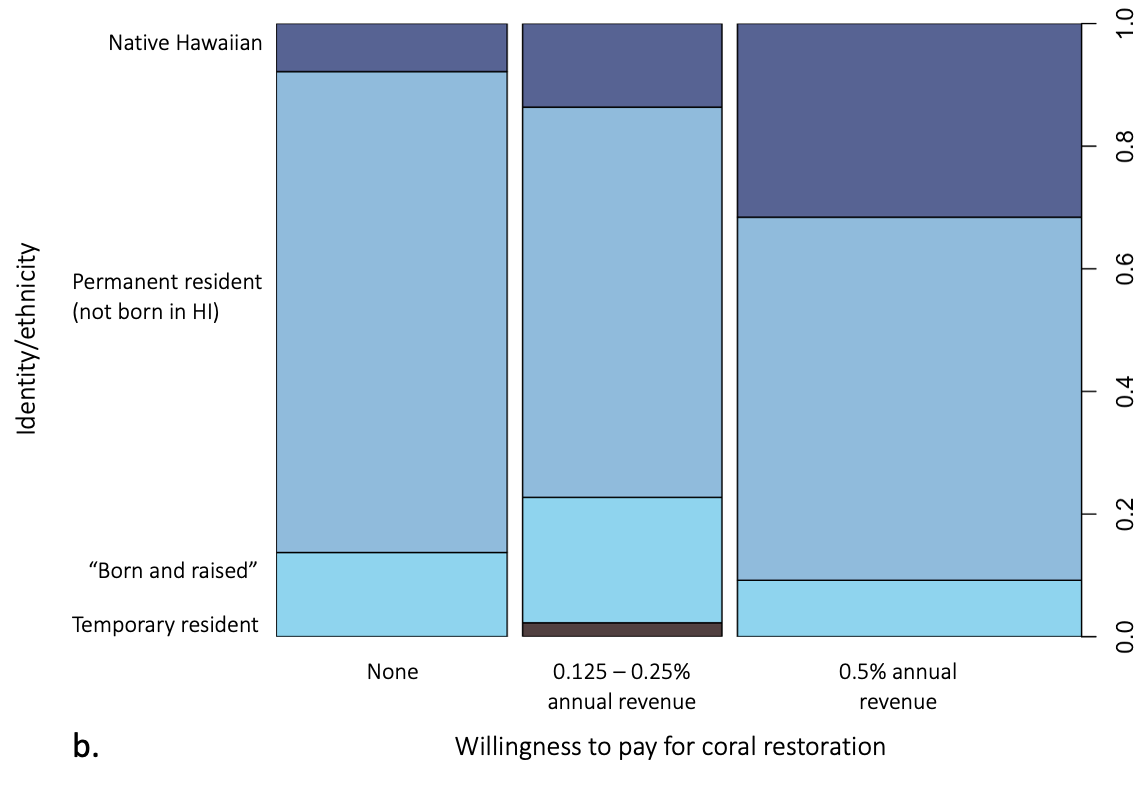


Share of total responses

**Figure S2:** Distribution of **a)** seniority (years with company) and **b)** Hawaiʻi residence and ethnicity across maximum WTP levels. The x-axis represents the highest payment level each respondent was willing to accept, while the y-axis represents the share of respondents within each demographic category.


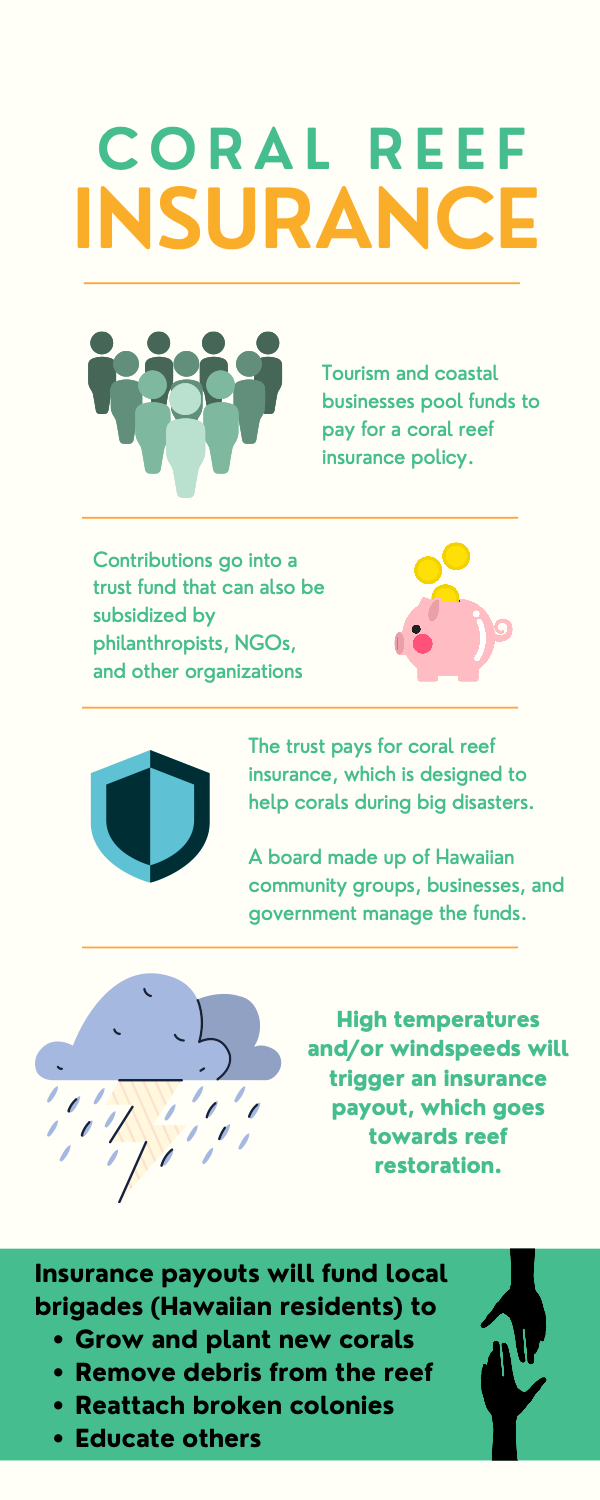


**Figure S3:** Explanation of coral insurance program to fund coral restoration provided to survey participants.
